# A rapid sensitive, flow cytometry-based method for the detection of *Plasmodium vivax*-infected blood cells

**DOI:** 10.1186/1475-2875-13-55

**Published:** 2014-02-14

**Authors:** Wanlapa Roobsoong, Steven P Maher, Nattawan Rachaphaew, Samantha J Barnes, Kim C Williamson, Jetsumon Sattabongkot, John H Adams

**Affiliations:** 1 Department of Global Health, College of Public Health, University of South Florida, Tampa, FL, USA; 2 Mahidol Vivax Research Unit, Faculty of Tropical Medicine, Mahidol University, Bangkok, Thailand; 3 Department of Biology, Loyola University, Chicago, IL 60660, USA

**Keywords:** Malaria, Vivax malaria, *Plasmodium vivax*, Flow cytometry, Diagnosis

## Abstract

**Background:**

*Plasmodium vivax* preferentially infects Duffy-positive reticulocytes and infections typically have few parasite-infected cells in the peripheral circulation. These features complicate detection and quantification by flow cytometry (FC) using standard nucleic acid-based staining methods. A simple antibody-based FC method was developed for rapid parasite detection along with simultaneous detection of other parasite and erythrocyte markers.

**Methods:**

Clinical samples were collected from patients diagnosed with *P. vivax* at a district Malaria Clinic in Kanchanaburi, Thailand. One μL of infected blood was washed, fixed, stained with a *Plasmodium* pan-specific anti-PfBiP antibody conjugated with Alexa Fluor 660, and analysed by FC. Additional primary conjugated antibodies for stage-specific markers of *P. vivax* for late trophozoite-early schizonts (MSP1-Alexa Fluor 660), late-stage schizonts (DBP-Alexa Fluor 555), and sexual stages (Pvs16) were used to differentiate intra-erythrocytic developmental stages.

**Results:**

The percentages of *P. vivax*-infected cells determined by the FC method and manually by microscopic examination of Giemsa-stained thick blood smears were positively correlated by Spearman’s rank correlation coefficient (*R*^*2*^ = 0.93843) from 0.001 to 1.00% *P. vivax*-infected reticulocytes.

**Conclusions:**

The FC-based method is a simple, robust, and efficient method for detecting *P. vivax*-infected reticulocytes.

## Background

Diagnosis of malaria parasites in field isolates typically relies on light microscopic detection of *Plasmodium*-infected blood cells in Giemsa-stained smears. Infection levels are often low and accurate determination of a parasitaemia requires the use of thick Giemsa-stained blood smears instead of thin blood smears used in acute infections or for monitoring *in vitro* cultures. Direct light microscopic observation *Plasmodium*-infected reticulocytes in thick smears is a slow procedure that is reliant on a skilled microscopist to manually count infected reticulocytes, since the parasites’ morphologic features and staining patterns are distorted in thick smears. The difficulty of accurate identification and quantification is exacerbated with *Plasmodium vivax*, which is a leading cause of malaria in many countries [1], because parasitaemias are relatively low and the variable properties of the host reticulocytes [2,3].

In recent years numerous flow cytometric-based methods have been developed to detect and quantify *Plasmodium falciparum* in the laboratory [4-9]. Many of these methods use nucleic acid staining to detect parasite-infected cells, since nucleated white blood cells (WBCs) are removed for culture and mature erythrocytes retain virtually no nucleic acid. Gradually these methods are being adapted for use in field laboratories and clinics. However, blood samples directly from malaria patients frequently have many contaminating WBCs and anaemia in chronic infections can enhance reticulocytaemia. Therefore, analysis of clinical isolates and the preference of *P. vivax* for reticulocytes, which still retain abundant amounts of nucleic acids in the cytosol, complicates the use of the nucleic acid stain-based flow cytometry (FC) methods. This has led to investigation of alternative staining strategies to identify the parasite-infected blood cells from field isolates. The objective of the study was to develop a strategy for rapid antibody-based staining to identify *P. vivax*-infected red blood cells as well as determine *P. vivax* stages of development. To identify parasite-infected blood cells, antibody to a C-terminal peptide epitope of PfBiP was used, which is a conserved cytoplasmic protein involved in endoplasmic reticulum (ER) retrograde trafficking [10,11]. Also known as the 78-kDa glucose regulated protein and heat shock protein 70–2, it is highly conserved amongst eukaryotic organisms [12]. Constitutive expression of BiP through asexual blood-stage development coinciding with continued ER development has led to the common use of anti-PfBiP as reference antigen in *P. falciparum* studies [13,14]. The antibodies to this conserved ER-resident protein were reactive with *P. vivax* and quantification of parasite-infected reticulocytes correlated accurately with light microscopic calculations.

## Methods

### Fresh isolates human malaria parasites

The Ethical Review Committee of Faculty of Tropical Medicine, Mahidol University, approved a Human Subjects protocol for this study. Fresh isolates of *P. vivax* were collected from symptomatic patients attending the malaria clinics in Kanchanaburi Province in districts near the western border of Thailand. After informed consent was obtained, a 20 ml sample of *P. vivax*-infected blood was drawn by venipuncture into a 50 ml tube containing heparin. Thick and thin smears were made from 1 μl of packed blood cells before and after removal of WBCs. After being completely dried, thin blood film was fixed with absolute methanol for 30 sec. Both thick and thin blood films were stained with Giemsa at 1:10 dilution for 15 min, rinsed with water, allowed to dry completely, and the sample was blinded when counting by light microscopy.

### Short-term *in vitro* culture of *Plasmodium vivax*

WBCs were removed from *P. vivax*-infected blood by passage through Plasmodipur filter (EuroProxima) [15]. For short-term culture, filtered *P. vivax*-infected blood was incubated for 20–24 hr with McCoy’s 5A medium (Sigma) supplemented with 25% heat inactivated human AB serum (Interstate Blood Bank) in T75 cm^2^ tissue culture flask. The culture flask was placed in a sealed container purged under mixed gas 90:5:5% N:O:CO_2_ and maintained at 37°C [16].

## Indirect immunofluorescence assay (IFA)

### Preparing IFA microscope slides

Field isolates of *P. vivax* and a laboratory line of *P. falciparum* strain NF54 were used to prepare thin blood smears on microscope slides for IFA. Briefly, after 20-24-hr culture *P. vivax*-infected blood was separated by 47% Percoll (Sigma) gradient centrifugation and an enriched fraction of schizont-stage parasites were collected at the gradient interface [16]. After three washes with PBS the enriched parasite fraction was diluted to 1% (v/v) with PBS and 1 μl of diluted parasite was spotted on multi-well slides. *Plasmodium falciparum* NF54 IFA microscope slides were prepared from cultures (RPMI1640 medium supplemented with 5% Albumax to a 10% parasitaemia) concentrated by centrifugation, washed extensively with PBS, and suspended to 1% (v/v) with PBS. One μl aliquot of diluted parasite-infected reticulocyte suspension was spotted on multi-well slides, air dried at room temperature (RT), hermetically sealed, and stored at -20°C until used.

### IFA staining

Parasites were fixed with 4% paraformaldehyde for 20 min at RT, treated with 1% Triton X-100 (TX-100) for 20 min at RT, and washed three times with PBS containing Tween-20 (PBST). To minimize non-specific binding, IFA slides were treated with 3% BSA for 30 min at 37°C and washed 3 times with PBST. Finally, parasites were stained for 30 min at RT in a dark moist chamber with anti-PfBiP (MRA28) Alexa Fluor 660 at a 1:100 dilution alone and in combination with anti-DBP (3D10) Alexa Fluor 488 at 1:100 dilution or with anti-PvMSP1-19 Alexa Fluor555 at 1:100 dilution. For staining of gametocytes, parasites were stained with anti-pvs16 for 30 min at RT. After washing with PBST a combination of anti-PfBiP Alexa Fluor 660 at 1:100 diluion and Goat anti-mouse Alexa Fluor 488 at 1:500 dilution was added. After staining, microscope slides were washed three times, DAPI plus anti-fade was applied to the slide, and the slide was covered with a cover slip. Parasites were observed by epifluorescence and phase contrast microscopy (Olympus IX71 and DeltaVision CORE) and confocal microscopy (Zeiss LSM700). Micrograph images were prepared for publication using SoftWorx (Applied Precision) and ZEN (Zeiss).

### Direct conjugation of antibodies

Total IgG was purified from anti-PfBiP [11] rabbit sera (MRA28) and anti-PvMSP1-19 (MRA30) rabbit sera using a Protein G column (HiTrap Protein G HP, GE Healthcare). One milligram of purified IgG from each antibody was directly conjugated with Alexa Fluor fluorescence dye (Molecular Probe) according to manufacturer protocol. Anti-PfBiP purified rabbit IgG was conjugated with Alexa Fluor 660, anti-PvMSP1-19 purified rabbit IgG was conjugated with Alexa Fluor 555, and anti-DBP (3D10) monoclonal antibody was conjugated with Alexa Fluor 488. All direct conjugated antibodies were aliquot and kept at -20°C.

### Sample preparation for flow cytometry

One μL of infected blood was suspended in 100 μL of wash buffer PBS-B (PBS + 0.05% BSA) and fixed with 100 μL of 0.05% glutaraldehyde for 5 min at RT. Post fixation, cells were washed in PBS-B, treated with 100 μL of 0.3% TX-100 for 5 min at RT, washed twice in PBS-B, and blocked with 3% BSA for 5 min at RT. After washing in PBS-B, samples were processed for antibody staining at RT for 10 min in the dark, using 100 μL of the following: (i) anti-PfBiP Alexa Fluor 660 (1:100); (ii) anti-PvMSP1-19 Alexa Fluor 555 (1:100) (iii) anti-DBP (3D10) [17] Alexa Fluor 488; and, (iv) anti-Pvs16 (1:100) (mouse antiserum prepared against bacterial-expressed product) followed by secondary antibody staining (washed 1X PBS-B and then incubated with 100 μL of goat anti-Mouse IgG FITC (1:1,000) (DAKO)) for 10 min at RT in the dark). After washing with 100 μL of PBS + 0.05% BSA, the sample was suspended with 400 μL of PBS-B. Samples (ii), (iii) and (iv) were co-stained with anti-PfBiP Alexa Fluor 660 (1:100). As a control, 1 μl of packed uninfected blood, without removal of WBCs, was stained along with the infected blood and used as the control. The samples were kept in the dark at 4°C until FC analysis.

### Flow cytometry

The analysis by FC was performed on an Accuri C6 (BD Biosciences) with standard optic configuration (488 nm blue laser and 633 nm red laser). The threshold was set at 80,000 on forward scatter (FSC-A). The sample was transferred to 12*75 mm round bottom tube. One million events were acquired for each sample. Data analysis was performed with CFlow Sampler version 1.0.227.4 (BD Biosciences).

### Statistical analysis

A Spearman correlation was performed, using SAS 9.2 (Cary, NC, USA released 2008), to determine if there was a correlation between the parasitaemia calculated by microscopy and the parasitaemia calculated by flow cytometry.

## Results

### *Plasmodium* BiP is conserved

**Figure 1:**
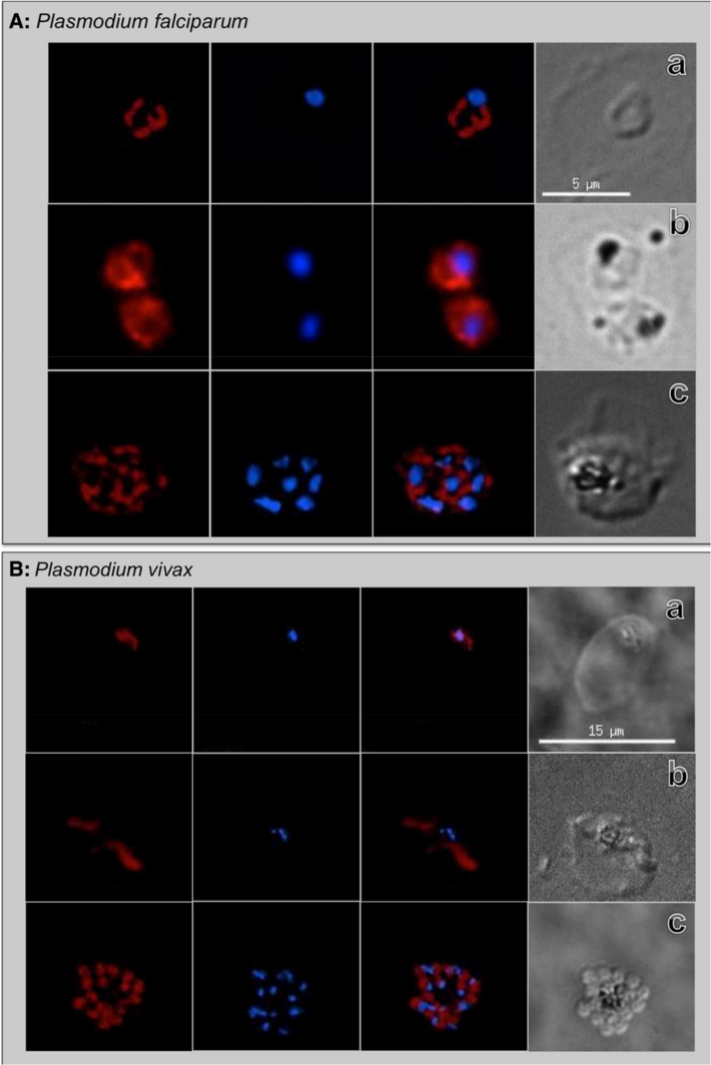
**Reactivity of anti-PfBiP Alexa Fluor 660 to (A) *Plasmodium falciparum *ring, trophozoite and schizont stage and (B) *Plasmodium vivax *blood-stage parasites.** Reactivity to ring, trophozoite and early schizont stage had a dispersed cytoplasmic fluorescence pattern **(a-b)** and in late-segmented schizonts was concentrated at the apical end of daughter merozoites **(c)** of both species.

*Plasmodium falciparum* BiP is an abundantly expressed protein of the ER involved in retrograde transport. Previous studies identified PfBiP as a useful localization marker and as a reference standard loading control for analysis of relative levels of parasite protein expression [11,18,19]. The IFA results in this study reconfirmed these observations demonstrating this anti-PfBiP reacted with all intra-erythrocytic developmental stages of *P. falciparum* (Figure 1A). Localization patterns reflected temporal and spatial changes in localization consistent [with] morphology of the ER during asexual stage growth and development. The fluorescence signal was generally dispersed in the cytoplasm of ring-stage parasites, increasing in abundance during trophozoite and early schizont stages before concentrating in the perinuclear apical end of forming merozoites in late segmenting schizonts.

A comparison of the *P. falciparum* BiP peptide sequence (SGDEDVDSDEL of PF3D7_0917900) that was used to produce the anti-PfBiP antibodies to the orthologous *P. vivax* sequence (SADEDVDSDEL of PVX_099315) indicates that this sequence is highly conserved (Table 1). To investigate usefulness of anti-PfBiP as a marker for *P. vivax*, this antibody was tested for IFA reactivity to intra-erythrocytic developmental stages of *P. vivax*. The fluorescence signal was similar to the dispersed patterns in the cytoplasm of ring, trophozoite and early schizont when it is concentrated at the apical end of daughter merozoites in segmented schizont of *P. vivax* (Figure 1B). The anti-PfBiP was specific for the cytoplasm of the *P. vivax* and did not react to uninfected reticulocytes.

**Table 1:** **Peptide sequences of BiP among *Plasmodium *** **species**

***Plasmodium *** **species**	**Gene ID**	**Amino acid sequence**
*P. falciparum*	PF3D7_0917900	SGDEDVDSDEL
*P. vivax*	PVX_099315	SaDEDVeSDEL
*P. knowlesi*	PKH_071520	SGDEDVeSDEL
*P. yoelii*	PY05001	pGDEDVDSDEL
	Consensus	**DEDV*SDEL

BiP peptide sequences from *P. falciparum, P. vivax, P. knowlesi* and *P. yoelii* were compared. The concensus peptide sequence of BiP (**DEDV*SDEL) was identified from *P. falciprum* BiP orthologs. Sequences were obtained from PlasmoDB (version 10.0) [20].

**Figure 2:**
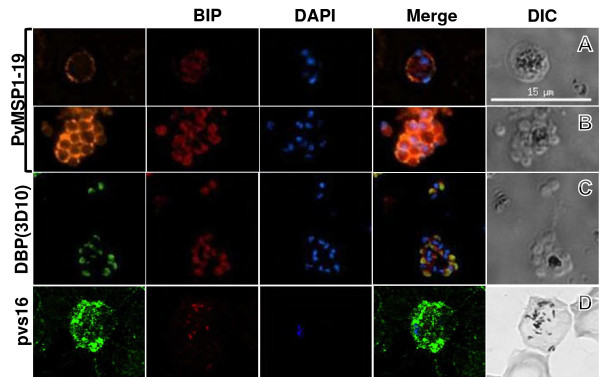
**Immuno co-localization of BiP with MSP1 and DBP in schizont stages of *Plasmodium vivax*. (A)** MSP1 is localized in close proximity to the plasma membrane of early schizonts and surrounding merozoite membranes in late-segmented schizonts. **(B)** BiP is distributed throughout the cytoplasm of early schizonts and concentrated at the apical end of the merozoite in late segmenting schizonts. **(C)** In late segmenting schizonts, BiP is localized at the apical end in close proximity to the nucleus of daughter merozoites while DBP is localized at the apical end next to BiP. **(D)** Pvs16 was detected only in morphologic forms identified as gametocytes, localizing mainly in the peripheral cytoplasm.

Stage-specific antibodies anti-PvMSP1 and anti-DBP (3D10) were evaluated in combination with anti-PfBiP to distinguish *P. vivax* developmental stages. In trophozoite/early schizont stages, the anti-PvMSP1 showed the circular fluorescence pattern in close proximity to the parasite plasma membrane while anti-PfBiP showed the diffuse fluorescence pattern in the cytoplasm of the parasite (Figure 2). In late-stage segmented schizonts, anti-PfBiP showed fluorescence signal at the apical end in close proximity to the nucleus of the merozoite while the anti-PvMSP1 fluorescence pattern localized at the membrane surrounding the daughter merozoites. The anti-DBP (3D10) fluorescence was detected only in late schizonts and localized at the apical end immediately adjacent to BiP localization (Figure 2C). In *P. vivax* gametocytes, the anti-Pvs16 was widely distributed in the peripheral cytoplasm while anti-BiP staining was relatively less in a dispersed punctate pattern (Figure 2D).

### Flow cytometry gating parameters

**Figure 3:**
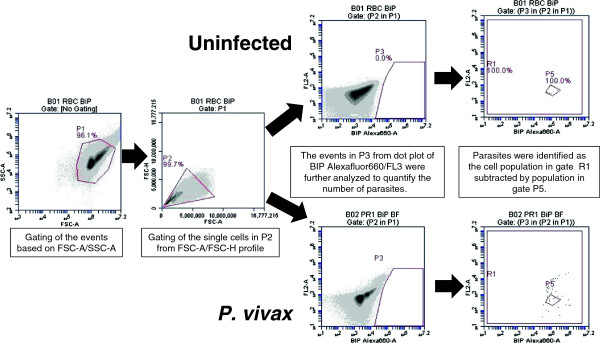
**Gating strategy for BiP + events.** Uninfected erythrocytes were used as a control for gating purpose. The events were gated based on the forward scatter and side scatter (FSC-A/SSC-A) profile in the dot plot (P1). The events in gate P1 were viewed in a FSC-A/FSC-H profile to distinguish and select cell singlet in gate P2. The single cells from gate P2 were viewed in a BiP Alexa 660-A/ FL2-A dot plot. The events in gate P3 were selected and viewed in BiP Alexa 660-A/ FL2-A dot plot. The BiP + events were obtained by subtracting the events in gate R1 with the events in gate P5.

**Figure 4:**
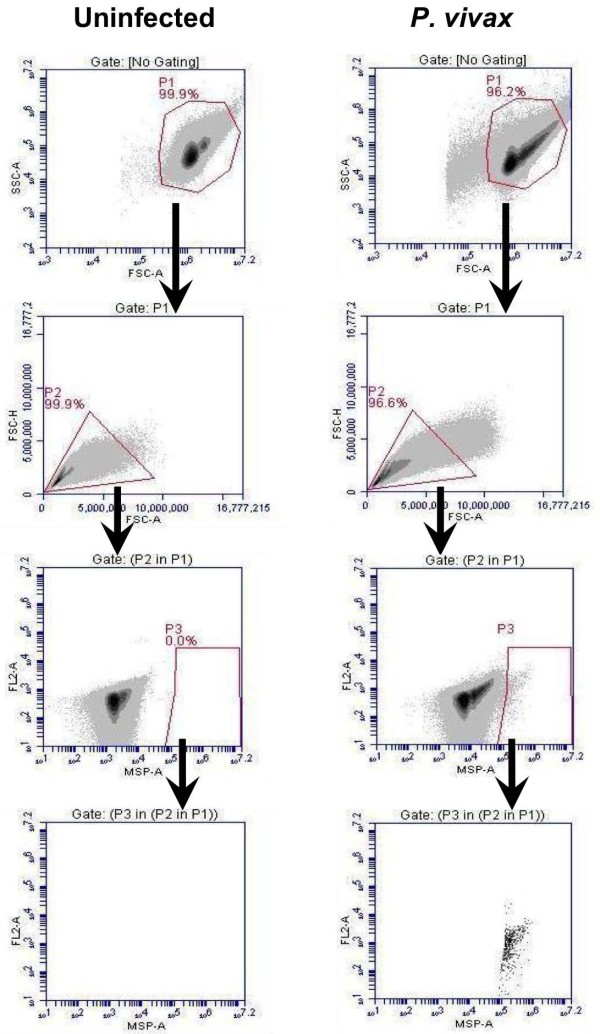
**Gating strategy for MSP-1 positive (MSP+) events.** The events in gate P1 were viewed in a FSC-A/FSC-H profile to distinguish and select cell singlet in gate P2. The single cells from gate P2 were viewed in a MSP-A/ FL2-A dot plot. The events in gate P3 were identified as MSP + events.

**Figure 5:**
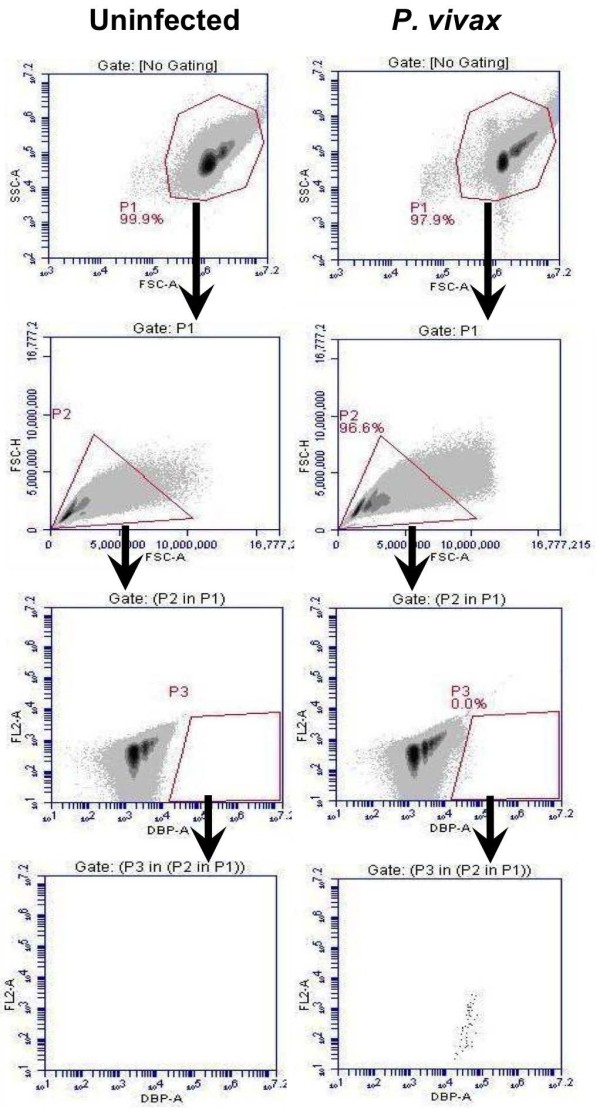
**Gating strategy for DBP positive (DBP+) events.** The events in gate P1 were viewed in a FSC-A/FSC-H profile to distinguish and select cell singlet in gate P2. The single cells from gate P2 were viewed in a DBP Alexa-A/ FL2-A dot plot. The events in gate P3 were identified as DBP + events.

**Figure 6:**
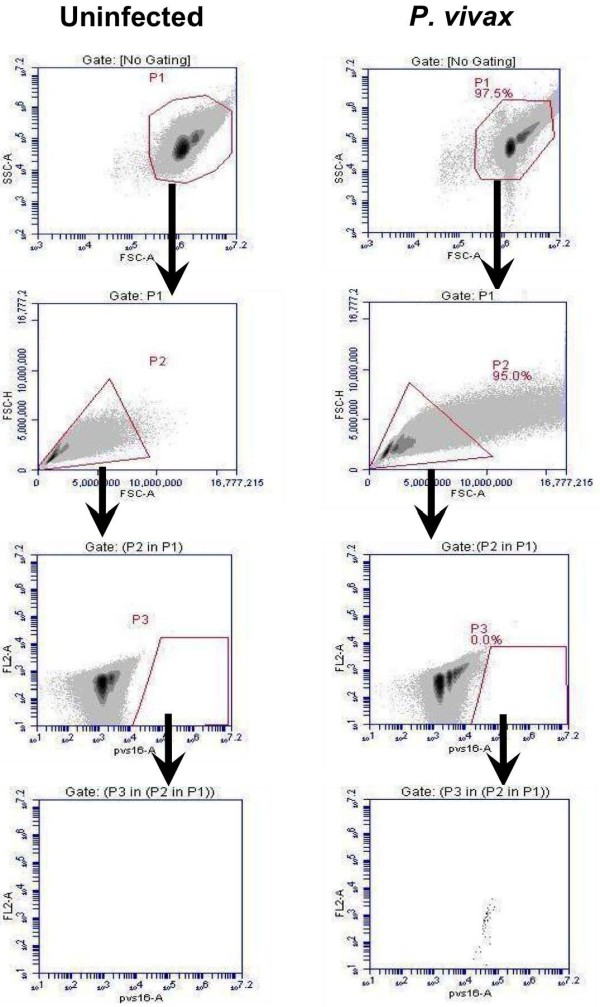
**Gating strategy for Pvs16 positive (Pvs16+) events.** The events in gate P1 were viewed in a FSC-A/FSC-H profile to distinguish and select cell singlet in gate P2. The single cells from gate P2 were viewed in a pvs16-A/ FL2-A dot plot. The events in gate P3 were identified as Pvs16+ events.

The rapid antibody IFA staining method using anti-PfBiP Alexa Fluor 660 was adapted to blood cells in suspension and evaluated by FC for specificity in detecting *P. vivax*-infected reticulocytes. In this analysis, Thai adult uninfected blood was stained with anti-PfBiP Alexa Fluor 660 for use as a negative control to define cell populations with non-specific or background reactivity (Figure 3). In the uninfected sample, events were gated based on the FSC-A/SSC-A profile in the dot plot (P1). The events in gate P1 were viewed in FSC-A/FSC-H profile to observe cell duplet. The single cells from gate P2 were selected to view in the anti-BiP Alexa 660-A/ FL2-A dot plot. The events in gate P3 were selected and viewed in anti-BiP Alexa 660-A/ FL2-A dot plot. In this plot, the events in gate P5 of the uninfected sample were considered as the background and were excluded from R1. In *P. vivax*-infected samples, parasites were identified as anti-BiP + events in gate R1 subtracted by the background in gate P5 (Figure 3). For identification of *P. vivax* late stages and gametocytes, the events were gated with the same strategy as for anti-BiP+, except the events were viewed in anti-MSP1 Alexa Fluor 555-A/ FL2-A (Figure 4), anti-DBP Alexa Fluor 488-A/ FL2-A (Figure 5) and anti-Pvs16-A/ FL2-A dot plot (Figure 6) for anti-PvMSP1-19, anti-DBP (3D10) and Pvs16 staining, respectively.

### Determination of parasitaemia and staging of the parasite

**Figure 7:**
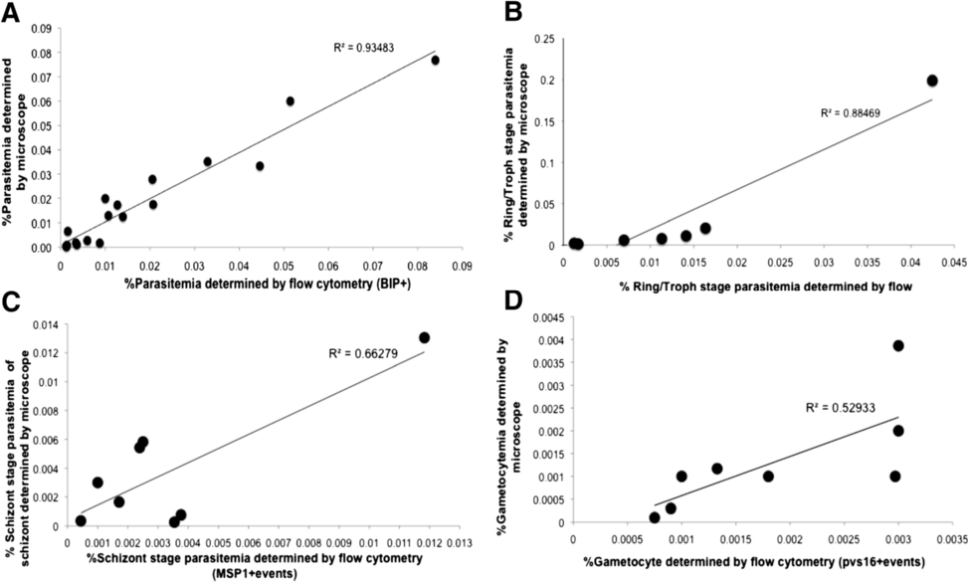
**Correlation of total parasitaemia (A), ring/trophozoite stage parasitaemia (B), schizont stage parasitaemia (C), and gametocytaemia (D) of *Plasmodium vivax *determined by flow cytometry and counting of the Giemsa-stained blood smear by light microscopy.** The total parasitaemia, ring/trophozoite stage parasitaemia, schizont stage parasitaemia and gametocytaemia obtained from flow cytometry shown positive correlation with the one obtained from counting of the Giemsa-stained blood smear by light microscopy (Spearman correlation coefficient = 0.9348 (N = 18), 0.8847 (N = 8), 0.6628 (N = 8) and 0.5293 (N = 8), respectively).

The FC parameters for anti-PfBiP Alexa Fluor 660 detection was used to estimate the total number of *P. vivax*-infected reticulocytes in all samples. In 18 clinical samples, parasitaemias were determined both by anti-PfBiP FC and by direct light microscopic observation of Giemsa-stained thick blood smears. There was a significant correlation of the parasitaemias determined by FC and microscopy (R^2^ = 0.94, P < 0.001, n = 18) (Figure 7A). To differentiate asexual developmental stages, anti-PfBiP was combined with stage-specific antibodies, anti-PvMSP1-19 and anti-DBP. MSP1-positive events identified stages beginning with mid-trophozoite/early schizonts through to completion of schizont development (MSP+). DBP-positive events were identified as beginning mid-schizont through to late-stage segmented schizonts (DBP+). Parasite stages positive for Pvs16 were identified as gametocytes (Pvs16+). Differential staining patterns were used to define the stages of development within each sample (Table 2). The number of ring and trophozoite stages was obtained by subtracting the number of BiP + events by the number of MSP1+ and Pvs16 + events while the number of early schizonts was obtained by subtracting the number of MSP1+ events by the number of DBP + events. The number of segmented schizonts and gametocytes were obtained directly from DBP + and Pvs16+ events, respectively. The parasitaemia of the individual asexual blood stages and the gametocyte-infected reticulocytes obtained from FC were compared with the corresponding values obtained from microscopy (Figure 7). There was a positive correlation of the ring stage parasitaemia (Figure 7B), schizont stage parasitaemia (Figure 7C) and gametocytaemia (Figure 7D) determined by FC and microscopy (R^2^ = 0.8847 (N = 8), 0.6628 (N = 8) and 0.5293 (N = 8), respectively).

**Table 2:** **Differential staining pattern of *P. vivax***

**Stage**	**BiP**	**MSP1**	**DBP**	**PVS16**
Ring to Mid-stage Trophozoite	+	-	-	-
Late-Trophozoite-early schizont	+	+	-	-
Mid-late Schizont	+	+	+	-
Gametocyte	+	-	-	+

Ring to mid trophozoite were stained with BiP, late trophozoite to early schizont were stained with BiP and MSP1, mid to late schizont were stained with BiP, MSP1 and DBP, and gametocyte was stained with BiP and pvs16.

**Figure 8:**
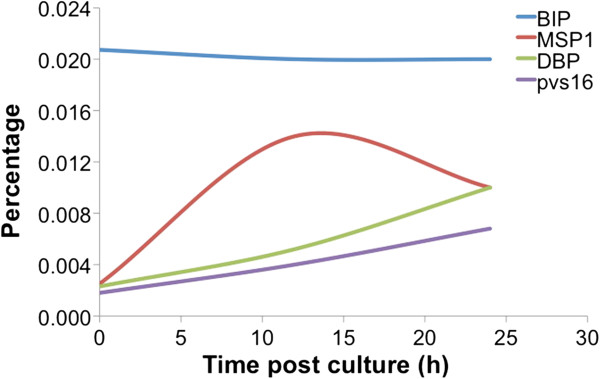
**Monitoring the development of the blood-stage *Plasmodium vivax *by antibody-based flow cytometry.** The number of MSP1+ and DBP + events increased while the number of BiP + events was stable during the examination. The number of pvs16+ events increased slightly during the culture period. The dynamic changes of MSP1+, DBP+, and Pvs16+ corresponded to the increasing number of schizonts, mature segmented schizonts and gametocytes, respectively.

To monitor the maturation of the parasites by flow cytometry, aliquots of *P. vivax*-infected blood was collected from short-term cultures at 12-hr and 24-hr intervals. The positive events from each stage-specific antibody were translated to maturation stage of the parasite (Table 2), using the strategy described above, and compared with those obtained from microscopic examination. There was an increase of MSP1+ and DBP + events while the number of BiP + events was similar during the examination period as determined by FC (Figure 8). Similar results were obtained by microscopic examination.

## Discussion

*Plasmodium falciparum* BiP is defined as an endoplasmic reticulum resident protein involved in retrograde transport. Important for this study, the peptide sequence of PfBiP used to prepare an anti-PfBiP peptide serum is very similar among *Plasmodium* orthologues. Previous studies have demonstrated that BiP is constitutively expressed during asexual blood-stage development and the sera reacted specifically to the ER compartment of *P. falciparum*, *Plasmodium berghei* and *Plasmodium yoelii*[11,21,22]. In this study, the broad pan-species reactivity of the anti-PfBiP was used to quantify *P. vivax* blood stages in clinical isolates and short-term *in vitro* cultures, demonstrating that the anti-PfBiP can be used as a universal antibody to detect developing blood stages of both *P. falciparum* and *P. vivax*.

Counting the parasitaemia by light microscopy is still the gold standard, but it is a difficult, time consuming method requiring a high degree of training to accurately identify *P. vivax* in thick Giemsa-stained blood smears. Flow cytometry is becoming a more accessible tool for many malaria research laboratories as the equipment becomes less expensive, more reliable and easier to use. Greater accessibility to FC has translated into more applications developed for *P. falciparum* research [23-25]. Most of the established methods for *P. falciparum* are based at least partly on nucleic acid staining. While these methods may be very useful for *P. falciparum* studies, they have been less valuable for *P. vivax* due to its preferential infection of reticulocytes that contain high levels of nucleic acids. The problem is compounded for analysis of *in vitro* and *ex vivo* cultures of *P. vivax* when reticulocytes derived from *in vitro* differentiation of hematopoietic stem cells are used and are contaminated with nucleated erythroid precursor cells.

## Conclusion

The antibody-based staining that has been developed in this study offers the opportunity for a robust, simple method for sensitive real-time detection of the *P. vivax*-infected cells in a standardized procedure that should reduce technical variation among researchers and laboratories. The reactivity of anti-PfBiP to both *P. falciparum* and *P. vivax* and any stages of blood-stage parasite offers an advantage of using this antibody for determining the parasitaemia by flow cytometry. With an optimized protocol, the antibody-based FC can detect one parasite per one million cells count (ten parasites/1 μl PRBC) and generates almost no background. When combined the anti-PfBiP with other stage specific antibodies, anti-MSP1-19, anti-DBP and anti-Pvs16, the antibody-based FC can be used to monitor the maturation of *P. vivax* parasite in the culture. The antibody based-FC using anti-PfBiP is a very sensitive and highly accurate method, which offers the new way to detect parasite in vivax malaria research.

## Abbreviations

BiP: Binding immunoglobulin protein of endoplasmic reticulum; DBP: Duffy binding protein; FC: Flow cytometry; IFA: Immunofluorescence assay; PvMSP1: *Plasmodium vivax* merozoite surface protein-1; PBS: Phosphate-buffered saline; PBST: PBS containing Tween-20; PfBiP: *Plasmodium falciparum* BiP; Pvs16: *Plasmodium vivax* sexual antigen 16; WBC: White blood cells.

## Competing interests

The authors declare that they have no competing interests.

## Authors’ contributions

WR and JHA conceived of the study. WR, SPM, JS and JHA designed the experiments. WR, SPM, NR, and SJB carried out the experiments. WR, SJB and JHA drafted the manuscript. All authors read and approved the final manuscript.
